# Hepcidin-Induced Iron Deficiency Is Related to Transient Anemia and Hypoferremia in Kawasaki Disease Patients

**DOI:** 10.3390/ijms17050715

**Published:** 2016-05-12

**Authors:** Ying-Hsien Huang, Ho-Chang Kuo, Fu-Chen Huang, Hong-Ren Yu, Kai-Sheng Hsieh, Ya-Ling Yang, Jiunn-Ming Sheen, Sung-Chou Li, Hsing-Chun Kuo

**Affiliations:** 1 Department of Pediatrics, Kaohsiung Chang Gung Memorial Hospital and Chang Gung University College of Medicine, Kaohsiung 833, Taiwan; erickuo48@yahoo.com.tw (H.-C.K.); huang817@adm.cgmh.org.tw (F.-C.H.); yuu2002@adm.cgmh.org.tw (H.-R.Y.); kshsieh@hotmail.com (K.-S.H.); e5724@adm.cgmh.org.tw (J.-M.S.); 2 Kawasaki Disease Center, Kaohsiung Chang Gung Memorial Hospital, Kaohsiung 833, Taiwan; 3 Department of Anesthesiology, Kaohsiung Chang Gung Memorial Hospital and Chang Gung University College of Medicine, Kaohsiung 833, Taiwan; yaling453@yahoo.com.tw; 4 Genomics and Proteomics Core Laboratory, Department of Medical Research, Kaohsiung Chang Gung Memorial Hospital and Chang Gung University College of Medicine, Kaohsiung 833, Taiwan; raymond.pinus@gmail.com; 5 Department of Nursing, Chang Gung University of Science and Technology, Taoyuan 33303, Taiwan; guscsi@gmail.com

**Keywords:** anemia, hepcidin, iron, Kawasaki disease

## Abstract

Kawasaki disease (KD) is a type of systemic vasculitis that primarily affects children under the age of five years old. For sufferers of KD, intravenous immunoglobulin (IVIG) has been found to successfully diminish the occurrence of coronary artery lesions. Anemia is commonly found in KD patients, and we have shown that in appropriately elevated hepcidin levels are related to decreased hemoglobin levels in these patients. In this study, we investigated the time period of anemia and iron metabolism during different stages of KD. A total of 100 patients with KD and 20 control subjects were enrolled in this study for red blood cell and hemoglobin analysis. Furthermore, plasma, urine hepcidin, and plasma IL-6 levels were evaluated using enzyme-linked immunosorbent assay in 20 KD patients and controls. Changes in hemoglobin, plasma iron levels, and total iron binding capacity (TIBC) were also measured in patients with KD. Hemoglobin, iron levels, and TIBC were lower (*p* < 0.001, *p* = 0.009, and *p* < 0.001, respectively) while plasma IL-6 and hepcidin levels (both *p* < 0.001) were higher in patients with KD than in the controls prior to IVIG administration. Moreover, plasma hepcidin levels were positively and significantly correlated with urine hepcidin levels (*p* < 0.001) prior to IVIG administration. After IVIG treatment, plasma hepcidin and hemoglobin levels significantly decreased (both *p* < 0.001). Of particular note was a subsequent gradual increase in hemoglobin levels during the three weeks after IVIG treatment; nevertheless, the hemoglobin levels stayed lower in KD patients than in the controls (*p* = 0.045). These findings provide a longitudinal study of hemoglobin changes and among the first evidence that hepcidin induces transient anemia and hypoferremia during KD’s acute inflammatory phase.

## 1. Introduction

Kawasaki disease (KD), an acute febrile systemic vasculitis, was initially described by Tomisaku Kawasaki in 1967. Its clinical diagnosis is largely based on a prolonged fever for at least five days and four or more of the following symptoms: conjunctivitis, enlargement of the cervical lymph nodes, oral mucosa changes, polymorphous skin rashes, and swelling of the hands and feet [1,2]. KD is the most common acquired heart disease in children among developed countries [1,2,3], with the most serious complication of coronary artery lesions (CALs), which have been found in 20%–25% of untreated children with KD [4,5].

As diagnosing KD currently relies on clinical signs, various nonspecific clinical symptoms can also manifest, such as irritability, uveitis, aseptic meningitis, pyuria, arthritis, hypoalbuminemia, impaired liver function, abdominal pain, hydrops of the gallbladder, anemia, and shock [5]. Among such manifestations, anemia is the most common in patients with KD and is believed to be caused by prolonged active inflammation [6,7,8]. Ling *et al.* [9] found that hemoglobin level was one of seven variables with the largest absolute values of coefficients in a dataset that consisted of 783 people, including 441 patients with KD and 342 febrile controls. Recently, some colleagues observed that anemia is also a useful early characteristic for differentiating KD shock syndrome from toxic shock syndrome in a pediatric intensive care unit [10]. However, the underlying cause of anemia in KD patients remains uncertain.

Hepcidin controls iron metabolism and the pathogenesis of anemia of inflammation [11]. In our previous study, we demonstrated that raised hepcidin levels are correlated with not only the development of anemia but also disease outcomes in patients with KD [12].The length that anemia and hypoferremia lasts in patients with KD is currently unknown. Therefore, we decided to study the hemoglobin levels in the different stages of KD. We then examined the correlations with plasma IL-6, hepcidin, and urine hepcidin levels, as well as red blood cell (RBC) parameters, including hemoglobin levels, mean corpuscular volume (MCV), and iron indices.

## 2. Results

### 2.1. Patient Characteristics

Of the 100 KD patients and 20 healthy control subjects enrolled in this study, 61 patients (61%) and 14 controls (70%) were male (*p* = 0.448). The mean ages of the patients with KD and the controls were 18.9 ± 1.6 and 17.4 ± 1.6 months (*p* = 0.343), respectively. With regard to treatment, 91 patients (91%) received one dose of IVIG, while the other nine patients (9%) received two. Echocardiography images revealed that 28 patients (28%) had CALs throughout the entire course of the disease.

### 2.2. Hemoglobin and Iron Levels and TIBC in the Kawasaki Disease (KD) Patients and Controls

**Figure 1:**
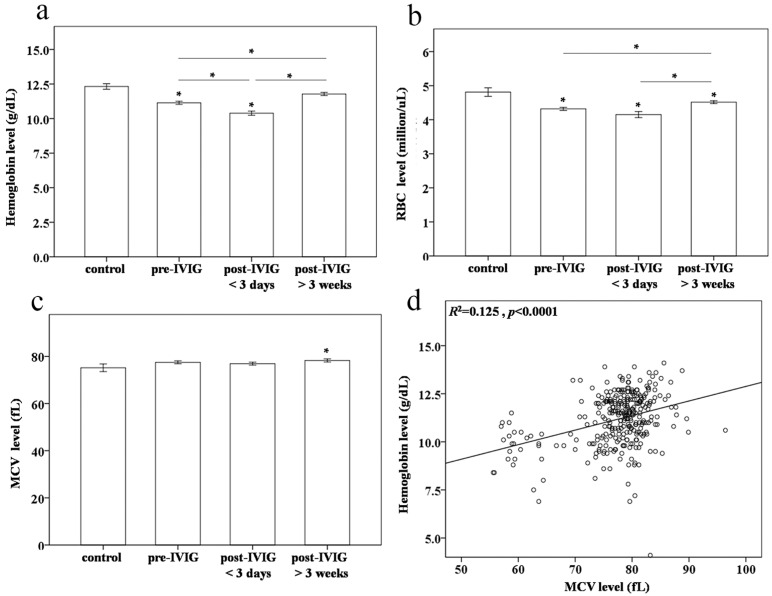
Comparison of (**a**) hemoglobin levels; (**b**) red blood cell (RBC) counts; and (**c**) mean corpuscular volume (MCV) between controls (*N* = 20), and patients with Kawasaki disease (KD) (*N* = 100) before and after undergoing intravenous immunoglobulin (IVIG) treatment; (**d**) Univariate analysis shows that the hemoglobin levels were positively and significantly correlated with MCV levels in both KD patients and the control subjects (*R*^2^ = 0.125, *p* < 0.0001). Data are presented as mean ± standard error. * indicates *p* < 0.05 between the control and KD groups.

The hemoglobin levels (11.1 ± 0.1 *vs.* 12.3 ± 0.2 g/dL, *p* < 0.001) and RBC counts (4.31 ± 0.04 *vs.* 4.81 ± 0.12 million/μL, *p*< 0.001) were lower in pre-IVIG KD patients than in controls, which is consistent with our previous findings (Figure 1) [12,13]. Furthermore, hemoglobin levels and RBC counts were considerably lower after IVIG treatment (10.4 ± 0.1 g/dL and 4.15 ± 0.09 million/μL, respectively, *p* < 0.001 and *p* = 0.01, respectively). In fact, a slight increase in hemoglobin levels was observed three weeks after IVIG treatment (*p* < 0.001) but was still lower than those in the controls (11.8. ± 0.1 g/dL, *p* = 0.045). Moreover, all MCV levels in post-IVIG >3 weeks KD patients were higher than those of the controls (*p* = 0.047) (Figure 1c). Univariate analysis demonstrated that the hemoglobin levels were positively and significantly correlated with MCV levels in both the KD patients and the control subjects (*R*^2^ = 0.125, *p* < 0.0001) (Figure 1d).

**Figure 2:**
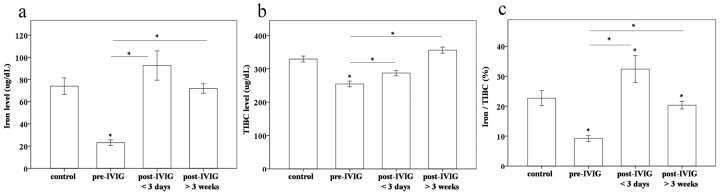
Comparison of (**a**) iron levels; (**b**) total iron binding capacity (TIBC); and the (**c**) iron/TIBC ratio between controls (*N* = 20) and patients with Kawasaki disease (KD) (*N* = 20) before and after undergoing intravenous immunoglobulin (IVIG) treatment Data are presented as mean ± standard error. * indicates *p* < 0.05 between the control and KD groups.

Then, we observed plasma iron levels and TIBC in 20 patients with various stages of KD and in 20 control subjects. Both the plasma iron levels and TIBC were lower in pre-IVIG KD patients (23.1 ± 2.5 and 254.4 ± 8.6 μg/dL, respectively) than in the controls (74.0 ± 7.5 and 329.1 ± 9.0 μg/dL, respectively, both *p* < 0.001),post-IVIG < 3 days KD patients (92.6 ± 13.1 and 287.1± 8.3 μg/dL, respectively, both *p* < 0.001), and post-IVIG > 3 weeks KD patients (71.8 ± 4.3 and 355.9± 9.2 μg/dL, respectively, both *p* < 0.001) (Figure 2a,b). In order to determine the percentage of transferrin saturation, we calculated the iron/TIBC ratio, which we found to be lowest in KD patients prior to undergoing IVIG treatment (*p* < 0.001 compared to the other groups) (Figure 2c).

### 2.3. Plasma and Urine Hepcidin Levels in The KD Patients and Controls

**Figure 3:**
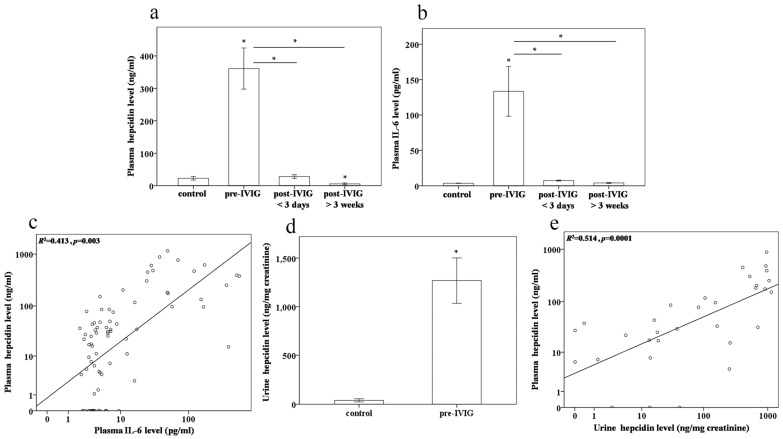
Comparison of plasma (**a**) hepcidin, (**b**) IL-6, and (**d**) urine hepcidin levels in patients with Kawasaki disease (KD) (*N* = 20) before and after undergoing intravenous immunoglobulin (IVIG) treatment; (**c**) Univariate analysis demonstrated that the log plasma hepcidin levels were positively and significantly correlated with the log IL-6 levels in patients with KD and the controls (*R*^2^ = 0.413, *p* = 0.003); (**e**) Univariate analysis demonstrated that the log plasma hepcidin levels were positively and significantly correlated with the log urine hepcidin levels in the KD before IVIG treatment and control groups (*R*^2^ = 0.514, *p* = 0.0001). Data are presented as mean ± standard error. * indicates *p* < 0.05 between the control and KD groups.

In our previous research, we found that prior to receiving IVIG treatment, plasma hepcidin and IL-6 levels were higher in patients with KD than in controls, and following IVIG treatment, plasma hepcidin and IL-6 levels were considerably reduced [12]. Plasma hepcidin and IL-6 levels were consistently higher in pre-IVIG KD patients than in the control subjects (all *p* < 0.001) (Figure 3a,b). Following IVIG treatment, plasma hepcidin and IL-6 levels were considerably lower (all *p* < 0.001), reaching their lowest values three weeks after patients underwent IVIG treatment. Univariate analysis revealed that the log plasma hepcidin levels were positively and significantly correlated with the log IL-6 levels in patients with KD and the controls (*R*^2^ = 0.413, *p* = 0.003, Figure 3c). Hepcidin is a peptide with a low molecular weight (2.78 kDa). After passing through the glomerular membrane, it is reabsorbed and degraded in the proximal tubules, with only a small fraction (3%–5%) of the filtered hepcidin passing intact into the urine [14]. The hepcidin levels in urine were higher in pre-IVIG KD patients than in controls (*p* < 0.001) (Figure 3d). Univariate analysis also demonstrated that the log plasma hepcidin levels were positively and significantly correlated with the log urine hepcidin levels in both pre-IVIG patients with KD and the control subjects (*R*^2^ = 0.514, *p* = 0.0001, Figure 3e).

### 2.4. Plasma Haptoglobin and Total Bilirubin Levels in the KD Patients and Controls

**Figure 4:**
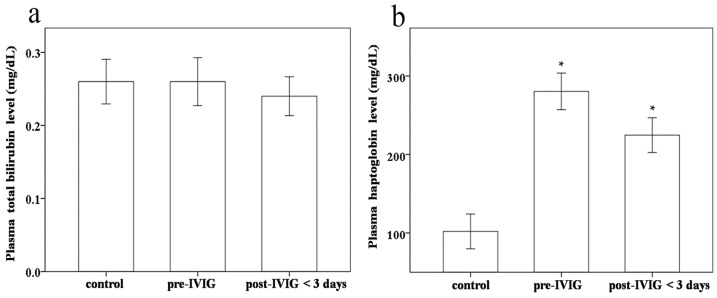
Comparison of (**a**) total bilirubin and (**b**) haptoglobin between patients with Kawasaki disease (KD) (*N* = 20) before and after undergoing intravenous immunoglobulin (IVIG) treatment and the control subjects (*N* = 20). Data are presented as mean ± standard error. * indicates *p* < 0.05 between the control and KD groups.

Some studies have found IVIG-related hemolysis in patients treated for KD [15,16]. Therefore, we studied the haptoglobin and total bilirubin levels as hemolysis markers between the groups. We found no significant difference in total bilirubin and haptoglobin levels between KD patients before and after receiving IVIG treatment (*p* = 0.621 and 0.075, respectively) (Figure 4a). We also know that haptoglobin is an acute phase protein [17,18]. An almost significant effect of downregulation of haptoglobin levels was consistently found in KD patients following IVIG treatment. Furthermore, we observed significantly higher haptoglobin levels in KD patients before and post-IVIG <3 days than in the control subjects (*p* < 0.001 and 0.002, respectively) (Figure 4b). Taken together, no definitive evidence of hemolysis was found in the participating KD patients following IVIG treatment.

## 3. Discussion

**Figure 5:**
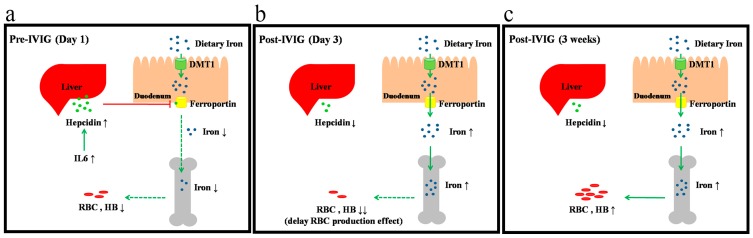
Proposed mechanism of hepcidin-induced hypoferremia and transient anemia in patients with Kawasaki disease. (**a**) Hemoglobin and iron levels were lower while plasma IL-6 and hepcidin levels were higher in patients with KD prior to IVIG administration; (**b**) After IVIG treatment, plasma hepcidin and hemoglobin levels significantly decreased; (**c**) There was a subsequent gradual increase in hemoglobin levels during the three weeks after IVIG treatment. The red solid line represents inhibition; the green solid line represents promotion; and the green dotted line represents reduction.

To the best of our knowledge, this study is among the first to provide a correlation that can explain the anemia seen in patients with KD. This anemia in KD is related to a markedly increased hepcidin expression that occurs due to a functional iron deficiency (Figure 5). We explained this phenomenon through a longitudinal study of the time-dependent changes in hemoglobin, hepcidin, and iron levels, as well as TIBC, particularly during KD’s convalescent stage. Furthermore, univariate analysis demonstrated that plasma hepcidin levels were positively and significantly correlated with urine hepcidin levels in pre-IVIG patients with KD.

Anemia is frequently seenin patients with KD due to the presence of prolonged active inflammation [6,7,8,19]. As shown in Table 1, we found that the affected inflammatory cytokines included IL-4 [20], IL-5 [20], IL-6 [12,21], IL-10 [21], IL-17A [21], and inducible protein-10 [22]. Inflammation-associated anemia represents a significant and highly prevalent clinical problem. Hepcidin plays a regulatory role in systemic iron homeostasis and serves as a mediator of host defenses and inflammation [23]. Increased hepcidin levels related to decreased iron bioavailability explain the pathogenesis of anemia with regard to acute and chronic inflammation. Hepcidin content has been demonstrated to increase to extremely high levels in patients after trauma, which positively correlates with the severity of injury and duration of anemia and negatively correlates with hypoxia [24]. Furthermore, high levels of hepcidin have also been observed in anemia associated with other inflammatory disorders, such as bacterial infections [25,26], autoimmune diseases [27,28], myocarditis, and myocardial infarction [24,29]. In our previous study, we found that anemia is related to a noticeably increased hepcidin expression [12]. Our most recent study also presented the novel observation that high-dose aspirin may also cause a lower hemoglobin level associated with a higher hepcidin level following IVIG treatment [13].

Hepcidin has a vital controlling influence on iron metabolism. Induced during infection and inflammation, it acts by binding to ferroportin, an iron exporter present on the absorptive surface of duodenal enterocytes, macrophages, and hepatocytes [30,31]. Upon such interaction, ferroportin is endocytosed and proteolysed, which subsequently leads to decreased iron absorption [32]. Ferroportin is currently the only known mammalian iron exporter [33]. All iron transfer to plasma occurs through ferroportin, is regulated by hepcidin, and is fine-tuned by regulatory mechanisms that serve iron homeostasis, host defenses, and erythropoiesis [33]. Furthermore, elemental iron, or iron not bound to protein, is taken up as Fe^2+^ by the H^+^/M^2+^ (DMT) symporter and DMT1 present on the luminal surface of enterocytes and macrophages [34,35]. Moreover, hepcidin both controls DMT1 [36] and directly inhibits erythropoiesis. Hepcidin has also been shown to inhibit erythroid progenitor proliferation and survival [37], which is consistent with the observation of transient erythroblastopenia following bone marrow aspiration in patients with KD [38]. In this study, hemoglobin levels dropped considerably following IVIG treatment, indicating that bone marrow suppression in patients with KD does not quickly reverse after IVIG treatment and a 3–5-day delay in the reticulocyte response for RBC production [39]. We have also studied the correlation between the hemoglobin levels and hepcidin in our previous study [12], in which the pre-IVIG hepcidin levels were negatively associated with the post-IVIG hemoglobin levels and positively associated with the differences of hemoglobin level (pre-IVIG levels minus post-IVIG levels) following IVIG treatment [12]. This agrees with our findings that hemoglobin levels immediately decreased considerably following IVIG treatment, with its levels gradually rising for at least three weeks after undergoing IVIG treatment. Plasma and urinary hepcidin levels were significantly correlated in our study, and we found that hepcidin is related to disease outcomes in KD patients [12]. A variety of body fluids, such as plasma and urine, are used in clinical analyses.Therefore, we suggest that the liquid biopsy of plasma andurinary hepcidin may be a useful non-invasive biomarker for prognosis and treatment response prediction of KD.

In fact, IVIG-related hemolysis has been found in patients treated for KD [15,16]. The major causes of hemolysis are generally associated with anti-A, anti-B IgM antibodies, and anti-Rh IgG antibodies. We used TBSF Human Immunoglobulin (Intragam^®^ P, CSL limited, Taiwan, China) in our KD patients, and this IVIG showed that at least 98% of the protein is IgG and contains very low titers of anti-A (1:8) and anti-B (1:4) IgM, and no anti-D IgG antibodies. Furthermore, Rh negative blood types are much less common in Asian populations (0.3%) than they are in Caucasian populations (15%) [40]. Therefore, the phenomena of hemolysis after IVIG in KD patients may be more commonly reported with regard to European ancestry than in Asian ancestry. However, future research would still need to check more meticulously the status of hemolysis, using such measurements as haptoglobin, bilirubin, reticulocyte count, and peripheral blood smear in KD patients following IVIG administration.

Since hepatocytes are the major producer of hepcidin and KD patients often present with hepatitis and jaundice [41], that may be one of the main causes of modified hepcidin expression in KD patients. Furthermore, Pinto *et al.* [42] suggested that an inappropriately low expression of hepcidin impairs normal lymphocyte proliferation. This raises the question of whether higher levels of hepcidin can control inflammation. An increasing amount of evidence has indicated that iron status is associated with coronary artery disease [43,44] and vasculitis [45,46]. Hepcidin plays an important role in iron metabolism and may play an indirect role in endothelial vasculitis. Therefore, some important issues remain to be investigated in KD patients.

The impact of iron deficiency in children is particularly important. For example, iron deficiency has been related to cognitive disturbances related to attention span, intelligence, and sensory perception [47]. Children with a chronic iron deficiency have demonstrated lower performance in language expression, environmental sound perception, and motor activity tasks than children with a normal nutritional iron status [48,49]. According to our results, plasma iron levels and TIBC returned to normal immediately after IVIG treatment, while hemoglobin levels remain significantly depressed. This phenomenon can be attributed to the time lag present in erythropoiesis. Notably, hemoglobin levels increased at three weeks after IVIG treatment, but the hemoglobin content still remained lower in patients with KD than in the control subjects. A longer follow-up period is necessary in these patients to fully understand the recovery of hemoglobin levels.

## 4. Patients and Methods

### 4.1. Patients

We enrolled a total of 100 patients with KD and 20 control subjects in this study. All patients were children that met the criteria for KD [5] and had received intravenous immunoglobulin (IVIG) treatment on the first day of KD diagnosis at Kaohsiung Chang Gung Hospital in Taiwan. The Institutional Review Board of Chang Gung Memorial Hospital provided its approval for this study, and informed consent was obtained in writing from the guardians of the participants. The patients were first treated with a single dose of IVIG (2 g/kg) during a 12-h period. Aspirin was also given as a single daily dose of 3–5 mg/kg, and patients continued to receive treatment until all signs of inflammation disappeared, as previously described in detail [12,50,51]. Furthermore, we obtained peripheral blood samples three times in accordance with a previous report [51]: prior to IVIG treatment (pre-IVIG) and within three days after finishing the initial IVIG treatment (post-IVIG < 3 days) to be the acute stage samples and at least three weeks after IVIG treatment to be the subacute stage samples (post-IVIG > 3 weeks). Such laboratory data as age, gender, RBC counts, hemoglobin, iron indices, IVIG treatment response, and CAL formation rate were collected for analysis inform the KD patients. For the purpose of this study, a CAL was considered a coronary artery internal diameter of at least 3 mm (4 mm if the subject was older than 5 years) or a segment with an internal diameter at least 1.5 times larger than that of an adjacent segment as seen through echocardiography [8,20]. IVIG responsiveness was characterized by the reduction of the fever (temperature > 38 °C) within 48 h of finishing IVIG treatment with no recurrence for at least seven days, along with the marked improvement or normalization of inflammatory signs [52]. Blood and urine samples were immediately placed in heparin-containing tubes, and the remaining aliquots of plasma were stored at −80 °C until used for analysis. The comparisons of plasma levels of hepcidin and IL-6 were measured in 20 patients of three stages with KD and the control subjects. Furthermore, we studied the urine hepcidin in 20 patients with KD before undergoing IVIG treatment and the control subjects.

### 4.2. Laboratory Measurements

#### Measurement of Cytokines Using Enzyme-Linked Immunoassay (ELISA)

The ELISA kits used for plasma IL-6 (human IL-6 Catalog Number: DY206, R&D Systems, Minneapolis, MN, USA) and the plasma and urine hepcidin-25 were commercially available competitive assays using synthetic hepcidin (Catalog Number: S-1337, Bachem Biosciences, St. Helens, UK, range: 0–25 ng/mL), and the methodology and performance characteristics have been described in a previous study [12]. The urine hepcidin quantity in each sample was normalized using urinary creatinine [53], and urinary hepcidin levels were expressed as nanograms of hepcidin per milligram of creatinine [54].

### 4.3. Statistical Analysis

All data are presented as the mean ± standard error. Quantitative data were analyzed using Student’s *t*-test, or when appropriate, one-way ANOVA. Furthermore, the least significant difference test was used for post-hoc testing when necessary. Changes before and after IVIG administration, as well as during the subacute stage, were evaluated with a paired-samples *t*-test, with *p* < 0.05 denoting statistical significance. All statistical tests were done with SPSS 22.0 for Windows XP (SPSS, Inc., Chicago, IL, USA).

## 5. Conclusions

Our results are the first to provide evidence showing that inflammation-induced hepcidin can induce transient anemia and hypoferremia in the acute phase of KD.

## Figures and Tables

**Table 1:** Cytokines expression between Kawasaki disease (KD) patients and controls.

Cytokines	KD Patients	Controls	*p*-Value
IL-4 [20]	12.07 ± 1.36 pg/mL (*N* = 95)	5.96 ± 0.54 pg/mL (*N* = 30)	<0.001
IL-5 [20]	5.17 ± 0.56 pg/mL (*N* = 95)	2.65 ± 0.55 pg/mL (*N* = 30)	<0.001
IL-6 [12,21]	152.29 ± 21.94 pg/mL (*N* = 110)	38.63 ± 12.40 pg/mL (*N* = 30)	<0.001
IL-10 [21]	109.79 ± 14.81 pg/mL (*N* = 110)	17.56 ± 7.88 pg/mL (*N* = 30)	<0.001
IL-17A [21]	25.35 ± 3.21 pg/mL (*N* = 110)	7.78 ± 1.78 pg/mL (*N* = 30)	<0.001
CXCL10 (inducible protein-10) [22]	3587 ± 210.2 pg/mL (*N* = 77)	921 ± 106.2 pg/mL (*N* = 77)	<0.001
